# Amino acid variants in the HLA-DQA1 and HLA-DQB1 molecules explain the major association of variants with relapse status in pediatric patients with steroid-sensitive nephrotic syndrome

**DOI:** 10.1186/s13052-025-01913-z

**Published:** 2025-03-14

**Authors:** Hui Yin, Sijie Yu, Xuelan Chen, Haiping Yang, Mo Wang, Qiu Li, Han Chan

**Affiliations:** Department of Nephrology, Children’s Hospital of Chongqing Medical University, National Clinical Research Center for Child Health and Disorders, Ministry of Education Key Laboratory of Child Development and Disorders, Chongqing Key Laboratory of Pediatric Metabolism and Inflammatory Diseases, Chongqing, 400014 China

**Keywords:** Idiopathic nephrotic syndrome, Steroid-sensitive nephrotic syndrome, Frequently relapsing, Steroid dependent, Human leukocyte antigen

## Abstract

**Background:**

Management of patients with steroid-sensitive nephrotic syndrome (SSNS) is challenging because of frequent relapses. Causal variants in the human leukocyte antigen (HLA) class II region that are associated with relapse remain undetermined.

**Methods:**

We collected a cohort of East Asian individuals comprising 206 pediatric patients with SSNS and 435 healthy controls from Southwest China. Ninety children with steroid-sensitive nephrotic syndrome without relapse (SSNSWR) and 116 children with steroid-dependent and/or frequent relapse nephrotic syndrome (SDNS/FRNS) were genotyped using Sanger sequencing. We then measured the transcriptional level, allele expression imbalance (AEI) and functional proteins of *HLA-DQA1* and *HLA-DQB1* in different stages of SDNS/FRNS.

**Results:**

rs1464545187 in *ANKRD36* was associated with an approximately 1.69-fold greater risk for SSNSWR (*P* = 0.04; 95% confidence interval [CI], 1.05–2.72). Clustered risk variants in *HLA-DQA1* and *HLA-DQB1* were significantly associated with SDNS/FRNS (rs1047989: *P* = 2.26E-07, odds ratio [OR] = 2.25, 1.65–3.05; rs9273471: *P* = 5.45E-05, OR = 1.84, 1.37–2.46; HLA-DQB1*06:02: *P* = 0.017, OR = 0.19, 0.04–0.77). The genotype distributions of rs1047989, 2:171713702, rs1049123, rs9273471, and HLA-DQB1*06:02 in patients with SSNS were significantly different from those in healthy controls. rs1047989 (HLA-DQA1) was significantly associated with a greater number of infections at relapse in SDNS/FRNS patients (*P* = 0.045, OR = 6.79, 95% CI: 1.29-168.52). Flow cytometry showed that the proportion of cells expressing HLA-DQA1^+^/DQB1^+^ (HLA-DQA1^+^, *P* = 0.0046; HLA-DQB1^+^, *P* = 0.0045) was lowest in the relapse stage. In addition, the mRNA levels of *HLA-DQA1* and *HLA-DQB1* were significantly greater in the relapse group than in the remission group (HLA-DQA1, *P* = 0.03; HLA-DQB1, *P* = 0.002). No significant AEIs were detected in the different stages of SDNS/FRNS. The rs1047989 variant is likely to affect the structure and stability of HLA-DQA1.

**Conclusion:**

rs1464545187 is a risk locus for SSNSWR but not SDNS/FRNS in Chinese children. Functional variations in *HLA-DQA1* and *HLA-DQB1* are implicated in regulating the immune response of SSNS patients, which may explain the typical triggering of SDNS/FRNS onset by infections.

**Supplementary Information:**

The online version contains supplementary material available at 10.1186/s13052-025-01913-z.

## Background

Idiopathic nephrotic syndrome (INS) is the most common childhood glomerular defect; INS is characterized by nephrotic-range proteinuria, a urinary protein/creatinine ratio ≥ 200 mg/mmol or a urine dipstick test showing 3 + protein, a serum albumin concentration < 30 g/L, and edema [1]. In the initial stage of treatment, patients receive a standard course of oral prednisone; most children respond within 4 weeks and are therefore diagnosed with steroid-sensitive nephrotic syndrome (SSNS) [2]. However, the management of patients with SSNS has been challenging because of frequent infections and high-dose steroid dependence, leading patients to progress to frequently relapsing nephrotic syndrome (FRNS) or steroid-dependent nephrotic syndrome (SDNS) [3]. SSNS patients who frequently relapse can develop life-threatening complications, including infection and thrombosis, hypovolemia, and acute kidney injury [4]. At present, there are no prognostic markers that enable an accurate prediction of FRNS or SDNS at disease onset.

The pivotal role of prednisone and the efficacy of immunosuppressive agents in SSNS treatment strongly implicate the immune system in the pathogenesis of this disease [5]. As an immune factor, the human leukocyte antigen (HLA) class II complex plays a critical role in the causal risk of developing SSNS. Most variants with associations identified by genome-wide association studies (GWAS) in SSNS are in the HLA-II region, which is associated with most autoimmune or infectious diseases. In 2014, Gbadegesin et al. [6] first conducted an exome array study and identified rs1129740 and rs1071630, which are located within HLA-DQA1, as candidate loci for SSNS in South Asian and White European patients. In 2018, Jia et al. [7] performed a GWAS with a replication study in a Japanese population and reported that the HLA-DR/DQ region is associated with childhood SSNS. One possible mechanism is that genes in the HLA class II protein complex and HLA class II receptor activity are strongly associated with the innate immune system, including the activation of professional cells (antigen-presenting cells and B cells), as well as the adaptive immune system, in which HLA molecules play a crucial role in the disease process [8]. The results of our recent GWAS suggested that the HLA-DQA/DQB region is likely strongly associated with disease relapse, and several risk single-nucleotide polymorphisms (SNPs) and HLA amino acid polymorphisms were associated with Chinese childhood SSNS, such as rs117962550, rs1464545187, rs1047989, 2:171713702, rs1049123, rs9273471, and HLA-DQB1*06:02; however, owing to the limited number of patients with SSNSWR and SDNS/FRNS, additional replication was not conducted [9]. To our knowledge, clinical and functional evidence on the associations between these SNPs and HLA-DQB1*06:02 and the molecular mechanism of HLA-DQA1 or HLA-DQB1 in Chinese SSNS patients has not been reported previously.

In this study, we sought to confirm the associations between genotypes and genetic models for these risk variants (rs117962550, rs1464545187, rs1047989, 2:171713702, rs1049123, and rs9273471) and HLA-DQB1*06:02 and SSNS in an independent Chinese population using Sanger sequencing. To further investigate the molecular mechanisms underlying disease relapse caused by HLA-DQA1 and HLA-DQB1, we collected data from a total of 196 Chinese patients with SSNS and 435 controls and detected the expression of HLA-DQA1 and HLA-DQB1, determined the allelic expression imbalance (AEI) of DQA1, and performed HLA structure modeling and molecular dynamic analysis.

## Methods

### Patients and study design

**Fig. 1:**
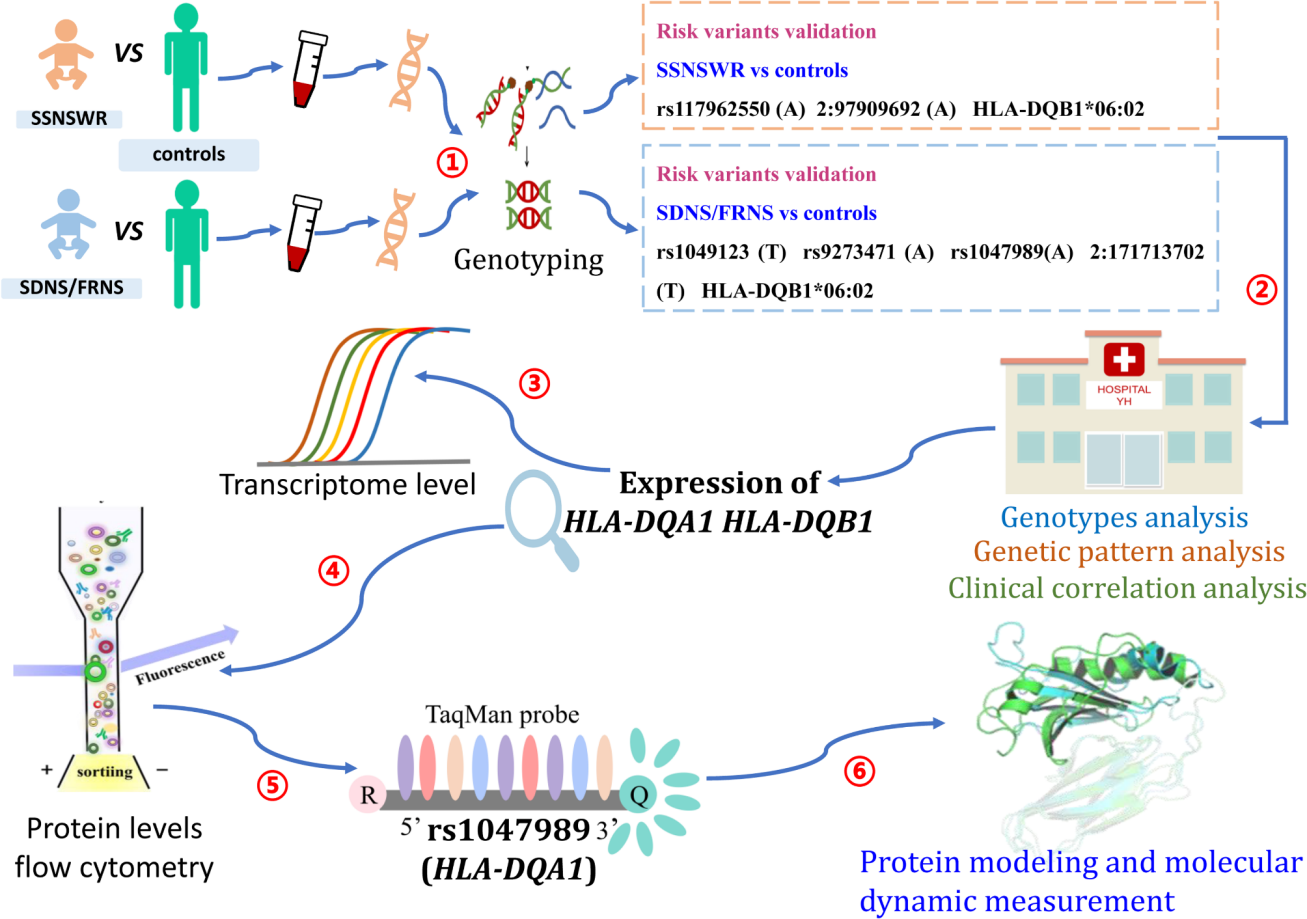
Flowchart of the study. The blue arrows represent the progression sequence of the study. The red circled numbers highlight the critical points of the study. 1: use of Sanger sequencing to separately validate the risk variant profiles in patients with SSNSWR and SDNS/FRNS. 2: clinical cohort analysis of patients carrying the risk variants, including genotype analysis, genetic pattern analysis, and clinical correlation analysis. 3 and 4: detection of RNA transcription levels and protein expression levels, respectively, of the *HLA-DQA1* and *HLA-DQB1* genes carried by the enrolled patients. 5 and 6: evaluation of the effect of the significant risk variant rs1047989 in patients with SDNS/FRNS on the allelic expression imbalance of the *HLA-DQA1* gene and assessment of its molecular structural stability, respectively. SSNSWR, steroid-sensitive nephrotic syndrome without relapse; SDNS/FRNS, steroid-dependent and/or frequent relapse nephrotic syndrome

The included patients with SSNSWR or SDNS/FRNS were aged 3 months to eighteen years and were recruited between July 2021 and June 2024. A total of 206 SSNS patients, including 90 with SSNSWR and 116 with SDNS/FRNS, who were admitted to the Department of Nephrology at the Children’s Hospital of Chongqing Medical University and 435 ethnicity- and sex-matched healthy controls from Southwest China were included in this study. All patients were enrolled according to strict inclusion and exclusion criteria (Table 1) [8, 10–11]. In clinical practice, a very small proportion of SSNS patients may be associated with genetic factors, especially in cases of familial clustering. For these patients, genetic testing can help identify pathogenic mutations related to podocytopathies. To ensure the representativeness of the SSNS patients included in our study, we reviewed the genetic testing results and excluded those with potential secondary steroid-resistant nephrotic syndrome (SRNS). During follow-up, the pediatric nephrologist categorized the patients based on their response to glucocorticoid treatment. The study was approved by the Ethics Committee of the Children’s Hospital of Chongqing Medical University (number 2020-40). Written informed consent was obtained from all the participating children and their guardians. A flowchart of the study design is shown in Fig. 1.

**Table 1:** Clinical criteria for inclusion and exclusion

	Subphenotypes of SSNS	
	SSNSWR	SDNS/FRNS
**Basic inclusion criteria**: 1. Age at disease onset was 3 months to 18 years. 2. Patients exhibited typical clinical manifestations and characteristics of SSNS but no significant extra renal manifestations. 3. There was no consanguinity among the patients.	**Inclusion criteria**: * Basic inclusion criteria. Adequate treatment with 2 mg/(kg·d) or 60 mg/(m^2^·d) prednisone resulting in proteinuria remission after 4 weeks of treatment. Withdrawal of SSNS patients from steroid treatment without relapse during the follow-up. Follow-up time of at least 12 months individually.	**Inclusion criteria**: *Basic inclusion criteria The following two conditions are met simultaneously: Two consecutive relapses during the reduction in corticosteroid therapy or within 2 weeks of the discontinuation of corticosteroid therapy. More than two relapses within a six-month period or relapse in more than 4 of twelve months.
**Exclusion criteria**:	1. Congenital nephrotic syndrome 2. Secondary nephrotic syndrome 3. Secondary SRNS 4. Patients with potential secondary SRNS (e.g., positive genetic test associated with podocytopathies)	

SSNS, steroid-sensitive nephrotic syndrome; SDNS/FRNS, steroid-dependent/frequent relapse nephrotic syndrome; SRNS, steroid-resistant nephrotic syndrome

### Baseline data collection and statistical analysis

The general characteristics of the patients, including demographic (e.g., age, sex, and ethnicity) and clinical data (e.g., relapse frequency and number of infections at relapse), were collected at the time of onset. Gene and genotype distributions were adjusted using the Haldane–Anscombe correction, and the chi-square test or Fisher’s exact test was used to calculate *P* values, odds ratios (ORs), and 95% confidence intervals (CIs) between groups. Logistic regression and FIRTH-modified logistic regression were employed. The measurement data were first subjected to normality and variance homogeneity tests. If the data were normally distributed and had equal variances, an independent sample t test was conducted; otherwise, non-parametric Wilcoxon rank-sum tests were used.

### Genotyping

The process of genomic DNA extraction is described in Supplementary Table 1. The gene polymorphisms were amplified using Sanger sequencing. The total polymerase chain reaction (PCR) mixture was 25 µl, including primers (1 µl), template DNAs (0.5 µl), high‑fidelity thermostable Taq enzymes (12.5 µl) and ddH_2_O (10 µl). The PCR conditions were as follows: 96 °C for 5 min; 45 cycles of 96 °C for 30 s, 56 °C for 45 s and 72 °C for 35 s; and 72 °C for 5 min. The primers used for polymorphic loci are listed in Supplementary Table 2.

### Measurement of HLA-DQA1 and HLA-DQB1 mRNA

The expression of HLA-DQB1 and HLA-DQA1 mRNA in peripheral blood mononuclear cells (PBMCs) was measured by quantitative reverse-transcription PCR. The samples from SSNSWR patients, SDNS/FRNS patients and healthy controls were derived from PBMCs that had been frozen in liquid nitrogen and stored in the biobank. According to the manufacturer’s instructions, total RNA was isolated from the PBMCs using an RNA rapid extraction kit (Accurate Biology, China). PCR was performed using cDNA as a template for the amplification of the HLA-DQB1 and HLA-DQA1 genes. The amplification reactions were quantified using the CFX96 system (Bio-Rad, USA). *GAPDH* was identified as the optimal internal reference gene in our study [12]. The gene-specific primers used for HLA-DQB1, HLA-DQA1, and GAPDH are listed in Supplementary Table 3.

### HLA-DQA1 and HLA-DQB1 expression on lymphocytes by flow cytometry

The PBMCs were thawed and resuspended in RPMI-1640 medium supplemented with 20% fetal bovine serum. Next, the cells were washed, and 5 µl of FC block (BioLegend, USA) was added according to the manufacturer’s instructions. The mixture was then incubated at 4 °C for 10 min in the dark. Next, according to the antibody titration results, 3 µl of DQA1 primary antibody (Proteintech, China) and 1 µl of DQB1 primary antibody (Abcam, USA) were added, and the samples were incubated for 30 min in the dark. Next, the cells were washed twice with cold phosphate-buffered saline (PBS). A donkey anti-mouse 488-conjugated fluorescent secondary antibody and a donkey anti-rabbit 647-conjugated fluorescent secondary antibody (Jackson, USA) were then added at a 1:300 dilution. After another two washes with cold PBS, the cells were resuspended in PBS and analyzed by flow cytometry.

### TaqMan PCR for HLA-DQA1 (rs1047989) typing

The rs1047989 locus of the HLA-DQA1 gene was investigated using the TaqMan fluorescent probe technique. The PCR mixture was prepared, consisting of 25µl in a 25 µl total volume, which included 0.6 µl of the probe (Accurate Biology, China), 0.5 µl of each forward and reverse primer, 12.5 µl of Probe qPCR Mix MultiPlus (Takara, Japan), 1 µL of DNA template (50 µg/µl), and 10.9 µl of nuclease-free water. For each sample, three replicates were prepared alongside positive and negative controls. The reaction mixture was aliquoted into 8-strip tubes, sealed, and then placed in a PCR apparatus for amplification. The allele type of the target gene in the samples was determined by evaluating the relative intensity of the fluorescence signals and the shape of the amplification curves. Samples were classified into three allele types: homozygous mutations (A/A), wild-type (C/C), and heterozygous mutations (A/C). The gene-specific primers for HLA-DQA1 were as follows: forward primer, 5’-AACACCAACTGCTGAGG-3’ and reverse primer, 5’-GCTCATCACGGTGGTC-3’. The corresponding probe sequences for the HLA-DQA1 gene included the wild-type probe FAM-TAAACAAAGCTCTGCTG-Eclipse (MGB) and the mutant probe FAM-TAAACAAAGCTCTGATG-Eclipse (MGB).

### TaqMan PCR revealed an AEI of DQA1

The TaqMan fluorescent probe technique was employed to detect AEI at the rs1047989 locus of the HLA-DQA1 gene in a disease group, and the results were compared with those of healthy control children who presented heterozygous mutations. Initially, the PBMCs were divided into two portions for the extraction of DNA and RNA. The cDNA and genomic DNA from the samples were then used as templates to formulate the reaction system. Three technical replicates were prepared for each sample, accompanied by positive and negative controls of known alleles. The reaction mixtures were aliquoted into eight tubes, sealed, and placed in a PCR apparatus for amplification. Two alleles of genomic DNA served as a 1:1 calibration reference to assess whether the ratio of the two alleles in the RNA of target tissue cells deviated from this expected ratio. We aimed to determine whether this ratio in the RNA of the target tissue cells was different from 1:1, indicating AEI and further elucidating the relationship between this locus and expression levels.

### Protein structure modeling and molecular dynamic measurement

AlphaFold3 was used to predict changes in protein structure [13]. Molecular dynamics (MD) simulation results, such as the root-mean-square deviation (RMSD), root-mean-square fluctuation (RMSF), radius of gyration (Rg), solvent accessible surface area (SASA) and dictionary of the secondary structure of protein (DSSP) were analyzed and computed using the g_rmsd, g_rmsf, g_hbond, g_gyrate, g_sasa and do_dssp built-in functions of GROMACS 4.5 [14].

## Results

### Characteristics of the study participants

The study cohort comprised 641 participants: 206 cases and 435 controls of East Asian descent recruited from a single university hospital in China. The overall male-to-female ratio was approximately 2:1, which is consistent with previous epidemiological studies. The median ages at diagnosis of the SSNSWR and SDNS/FRNS patients were 3.5 years (2.25–6.67 years) and 3.5 years (2.17–6.14 years), respectively. Considering that highcertainty evidence suggesting prolonged glucocorticoid treatment (> 12 weeks) increases the risk of adverse effects, especially severe infections caused by the treatment that could affect the experimental results, we have chosen 8–12 weeks of oral glucocorticoid treatment for the initial treatment of SSNS (Table 2).

**Table 2:** Characteristics of study participants

Variable	SSNSWR		SDNS/FRNS	
	Patients (*n* = 90)	Controls (*n* = 435)	Patients (*n* = 116)	Controls (*n* = 435)
Race	East Asian	East Asian	East Asian	East Asian
Male-to-female ratio	2.21:1	1.90:1	1.76:1	1.90:1
Median age at enrollment (range) (yr)	3.50 (2.25,6.67)	36.00 (29.00,42.00)	3.50 (2.17,6.04)	36.00 (29.00,42.00)

SSNSWR, steroid-sensitive nephrotic syndrome without relapse; SDNS/FRNS, steroid-dependent/frequent relapse nephrotic syndrome; NA, not applicable

### Risk variants associated with childhood SSNS in Chinese individuals

We identified 206 Chinese patients with childhood SSNS: 90 with SSNSWR and 116 with SDNS/FRNS. In the comparison between the SSNSWR group and the control group, one *ANKRD36* variant was significantly associated with SSNSWR (rs1464545187, *P* = 0.04). Carriers of the risk variant rs1464545187 had an approximately 1.69-fold greater risk for SSNSWR (odds ratio [OR], 1.69; 95% confidence interval [CI], 1.05–2.72). In the comparison between the SDNS/FRNS and control groups, three risk variants in HLA-DQA1 and HLA-DQB1 were significantly associated with SDNS/FRNS in Chinese children (rs1047989: *P* = 2.26E-07, odds ratio [OR] = 2.25, 1.65–3.05; rs9273471: *P* = 5.45E-05, OR = 1.84, 1.37–2.46; HLA-DQB1*06:02: *P* = 0.017, OR = 0.19, 0.04–0.77). These results confirmed that the HLA-DQA/DQB region is likely strongly associated with disease relapse, especially in SDNS/FRNS. In addition, there was a difference in the frequency of 2:171713702 between the SDNS/FRNS group and the control group (*P* = 2.2E-16). The details of the main risk SNPs at the respective loci are listed in Table 3.

**Table 3:** Candidate SNPs and HLA-DQB1*06:02 associated with Chinese SSNS

Candidate variants	rs117962550 (A)	rs1464545187 (A)	HLA-DQB1*06:02		
Gene	*ALPG*	*ANKRD36*	*HLA-DQB1*		
Frequency of risk allele in SSNSWR (*n* = 90)	9(5.00%)	26(14.44%)	5(2.78)		
Frequency of risk allele in controls (*n* = 435)	27(3.10%)	79(9.08)%	39(4.48%)		
*P* value	0.29	**0.04**	0.40		
OR (95% CI)	1.64(0.76,3.56)	1.69(1.05,2.72)	0.61(0.24,1.57)		
Candidate variants	rs1047989 (A)	2:171713702 (T)	rs1049123 (T)	rs9273471 (A)	HLA-DQB1*06:02
Gene	*HLA-DQA1*	*GAD1*	*HLA-DQB1*	*HLA-DQB1*	*HLA-DQB1*
Frequency of risk allele in SDNS/FRNS (*n* = 116)	158(68.10%)	71(30.60%)	16(6.90%)	117(50.43%)	2(0.86%)
Frequency of risk allele in controls (*n* = 435)	424(48.74%)	6(0.69%)	32(3.68%)	310(35.63%)	39(4.48%)
*P* value	**2.26E-07**	**2.2E-16**	0.05	**5.45E-05**	**0.01**
OR (95% CI)	2.25(1.65,3.05)	63.50(27.14,148.59)	1.94(1.05,3.60)	1.84(1.37,2.46)	0.19(0.04,0.77)

A, adenine; T, thymine; CI, confidence interval; G, guanine; OR, odds ratio; rs, reference single-nucleotide polymorphism identification number; SSNSWR, steroid-sensitive nephrotic syndrome without relapse; SDNS/FRNS, steroid-dependent/frequent relapse nephrotic syndrome

### Genotype distributions and genetic model analysis of risk variants

The genotype distributions of rs1047989 (*P* = 2.42E-06), 2:171713702 (*P* = 2.2E-16), rs1049123 (*P* = 9.75E-05), rs9273471 (*P* = 8.95E-06), and HLA-DQB1*06:02 (*P* = 0.02) in patients with SSNS were significantly different from those in healthy controls. These risk variants, rs1047989, rs1049123, rs9273471, and HLA-DQB1*06:02, in the HLA-DQA1/HLA-DQB1 region were associated with childhood SSNS. All three genetic models (recessive, dominant, and additive models) provided the best fit for rs1047989 and rs9273471. The dominant model and additive model provided the best fit for 2:171713702 and rs1049123. The details are shown in Table 4.

**Table 4:** Genotype distributions and genetic model analysis for risk variants

	Genotype	Case (%)	Control (%)	*P* value	OR (95% CI)
rs117962550 (A)					
Genotype	AA	0(0.00)	0(0.00)	0.1901	-
	AC	19(9.22)	27(6.21)		
	CC	187(90.78)	408(93.79)		
Recessive model	AA vs. (AC + CC)	0(0.00)/206(100.00)	0(0.00)/435(100.00)	-	**-**
Dominant model	(AA + AC) vs. CC	19(9.22)/187(90.78)	27 (6.21)/408(93.79)	1.69E-01	1.54(0.82, 2.82)
Additive model	AA vs. AC vs. CC	0(0.00)/19(9.22)/187(90.78)	0(0.00)/27 (6.21)/408(93.79)	1.70E-01	1.54(0.82,2.82)
rs1464545187 (A)					
Genotype	AA	0(0.00)	2(0.46)	0.40	-
	AG	42(20.39)	75(17.24)		
	GG	164(79.61)	358(82.30)		
Recessive model	AA vs. (AG + GG)	0(0.00)/206(100.00)	2(0.46)/433(99.54)	0.54	0.42(0.003,5.19)
Dominant model	(AA + AG) vs. GG	42(20.39)/164(79.61)	77(17.70)/358(82.30)	4.14E-01	1.19(0.78,1.80)
Additive model	AA vs. AG vs. GG	0(0.00)/42(20.39)/164(79.61)	2(0.46)/75(17.24)/358(82.30)	0.50	1.15(0.76,1.72)
rs1047989 (A)					
Genotype	AA	92(44.66)	113(25.98)	**2.42E-06**	-
	AG	89(43.20)	220(50.57)		
	GG	25(12.14)	102(23.45)		
Recessive model	AA vs. (AG + GG)	92(44.66)/114(55.34)	102(23.45)/333(76.55)	**7.53E-08**	2.63(1.85,3.75)
Dominant model	(AA + AG) vs. GG	181(87.86)/25(12.14)	322(74.02)/113(25.98)	**1.01E-04**	2.54(1.61,4.14)
Additive model	AA vs. AG vs. GG	92(44.66)/89(43.20)/25(12.14)	113(25.98)/220(50.57)/102(23.45)	**1.32E-08**	2.06(1.61,2.66)
2:171713702(T)					
Genotype	TT	0(0.00)	0(0.00)	**2.2E-16**	-
	CT	135(65.53)	6(1.38)		
	CC	71(34.47)	429(98.62)		
Recessive model	TT vs. (CT + CC)	0(0.00)/206(100.00)	0(0.00)/435(100.00)	**-**	-
Dominant model	(TT + CT) vs. CC	135(65.53)/71(34.47)	6(1.38)/429(98.62)	**2.14E-29**	135.95(62.68,356.99)
Additive model	TT vs. CT vs. CC	0(0.00)/135(65.53)/71(34.47)	0(0.00)/6(1.38)/429(98.62)	**2.14E-29**	135.95(62.68,356.99)
rs1049123 (T)					
Genotype	TT	0(0.00)	1(0.23)	**9.75E-05**	-
	CT	36(17.48)	30(6.90)		
	CC	170(82.52)	404(92.87)		
Recessive model	TT vs. (CT + CC)	0(0.00)/206(100.00)	1(0.23)/434(99.77)	0.8235151	0.70(0.004,13.21)
Dominant model	(TT + CT) vs. CC	36(17.48)/170(82.52)	31(7.13)/404(92.87)	**1.04E-04**	2.76(1.65,4.63)
Additive model	TT vs. CT vs. CC	0(0.00)/36(17.48)/170(82.52)	1(0.23)/30(6.90)/404(92.87)	**2.07E-04**	2.58(1.57,4.27)
rs9273471 (A)					
Genotype	AA	51(24.76)	64(14.71)	**8.95E-06**	-
	AG	104(50.49)	182(41.84)		
	GG	51(24.76)	189(43.45)		
Recessive model	AA vs. (AG + GG)	51(24.76)/155(75.24)	64(14.71)/371(85.29)	**2.18E-03**	1.91(1.26,2.88)
Dominant model	(AA + AG) vs. GG	155(75.24)/51(24.76)	246(56.55)/189(43.45)	**6.60E-06**	2.34(1.62,3.40)
Additive model	AA vs. AG vs. GG	51(24.76)/104(50.49)/51(24.76)	64(14.71)/182(41.84)/189(43.45)	**3.07E-06**	1.75(1.39,2.22)
HLA-DQB1*06:02					
Genotype	06:02/06:02	1(0.49)	4(3.22)	**0.02**	-
	06:02/Other	6(2.91)	35(8.05)		
	Other/other	199(96.60)	396(91.03)		

A, adenine; T, thymine; G, guanine; C, cytidine

### Correlation of genotype distributions with relapse frequency and infections at relapse in SDNS/FRNS patients

The clinical data for SDNS/FRNS patients were collected and used to identify the risk SNPs that potentially influence disease manifestations. The genotype distributions of the risk SNPs are presented in Table 5. rs1047989 (*HLA-DQA1*) was significantly associated with a greater number of infections at relapse in SDNS/FRNS patients (OR = 6.79, 95% CI: 1.29–168.52, *P* = 0.045), and the dominant model supported this result (*P* value = 0.02). In addition, the genotype distribution at 2:171713702 was associated with an increased risk of relapse for patients with SDNS/FRNS (OR = 2.59, 95% CI: 1.21–5.74; *P* = 0.02). The frequency of the glutamic acid decarboxylase 1 (*GAD1*) variant 2:171713702 differed between the disease group and the control group according to the dominant model (*P* = 0.02). This result is consistent with previously published studies [9]. No significant clinical associations were observed between the remaining SNPs and SDNS/FRNS.

**Table 5:** Correlation of genotypes with relapse frequency and infections at relapse in SDNS/FRNS patients

SNP	Clinical characteristics	Genotype frequency			*P*	Dominant model -*P* value	OR (95% CI)
rs1047989 (A)	Recurrence frequency (n/semiannual)	AA	AG	GG	0.67	0.56	0.60(0.17,1.96)
	2	27	23	8			
	>2	28	25	5			
	Number of infections at relapse	AA	AG	GG	**0.045**	**0.02**	6.79(1.29, 168.52)
	≤ 2	16	15	1			
	>2	39	28	17			
2:171713702 (T)	Recurrence frequency (n/semiannual)	TT	CT	CC	**0.02**	**0.02**	2.59(1.21, 5.74)
	2	0	42	16			
	>2	0	29	29			
	Number of infections at relapse	TT	CT	CC	0.12	0.17	2.13(0.84, 6.01)
	≤ 2	0	25	7			
	>2	0	46	28			
rs1049123 (T)	Recurrence frequency (n/semiannual)	TT	CT	CC	0.42	0.42	0.56(0.18,1.66)
	2	0	6	52			
	>2	0	10	48			
	Number of infections at relapse	TT	CT	CC	0.77	0.77	1.24(0.35,3.83)
	≤ 2	0	5	27			
	>2	0	11	73			
rs9273471 (A)	Recurrence frequency (n/semiannual)	AA	AG	GG	0.97	1	1.10(0.46, 2.65)
	2	14	31	13			
	>2	14	30	14			
	Number of infections at relapse	AA	AG	GG	0.07	0.07	3.03 (1.04,11.33)
	< median	7	32	4			
	> median	21	37	26			
DQB1:06:02	Recurrence frequency (n/semiannual)	DQB1:06:02 /DQB106:02	DQB1:06:02 /Other	Other /Other	0.50	0.62	0.35(0.01,3.14)
	2	0	0	58			
	>2	0	2	56			
	Number of infections at relapse	DQB1:06:02 /DQB106:02	DQB1:06:02 /Other	Other /Other	0.54	0.62	0.35(0.01,3.14)
	≤ 2	0	0	32			
	>2	0	2	56			

A, adenine; T, thymine; G, guanine; C, cytidine

### HLA-DQA1 and HLA-DQB1 are involved in SDNS/FRNS

**Fig. 2:**
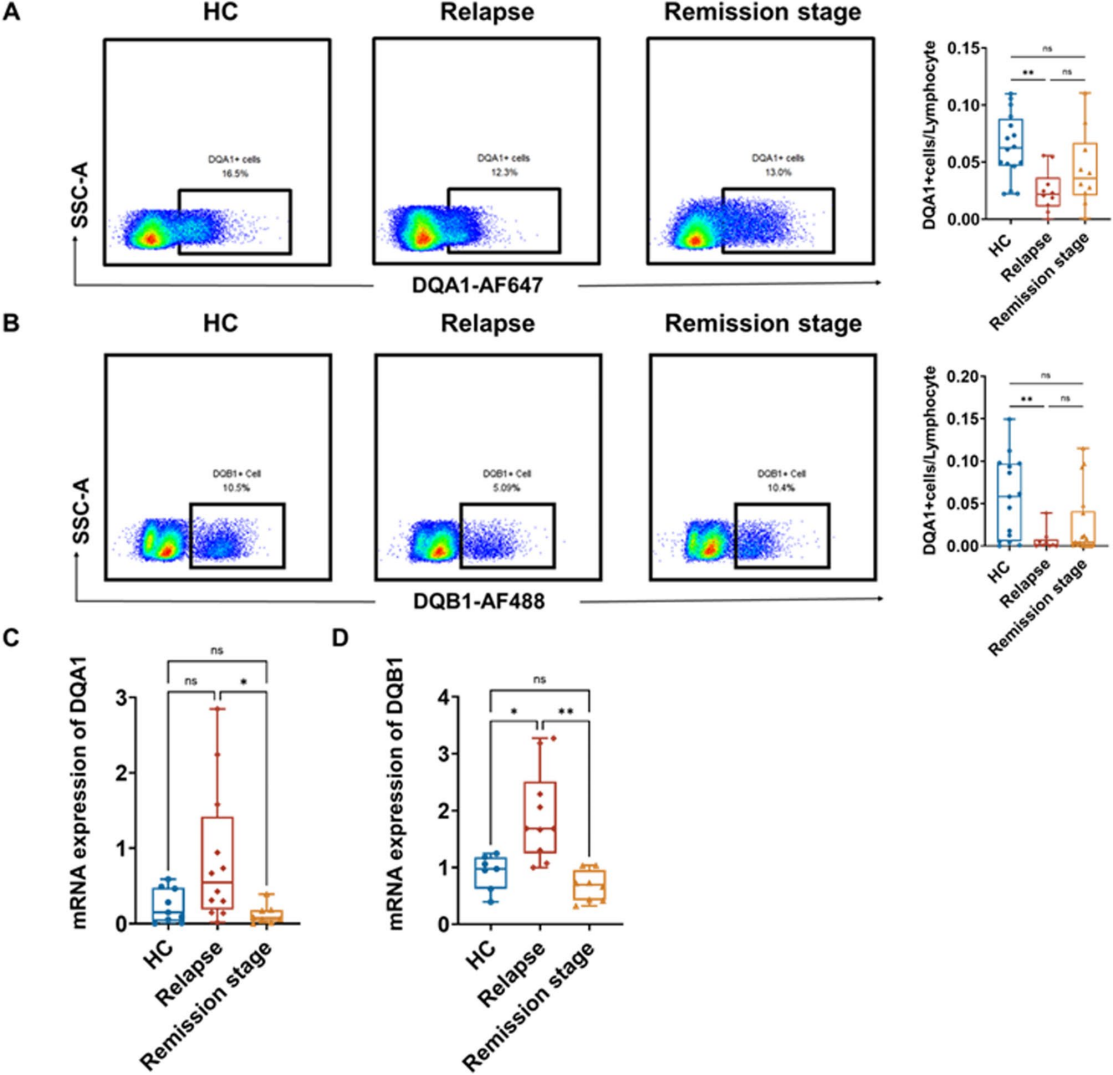
Transcriptional level and expression of *HLA-DQA1* and *HLA-DQB1* in different stages of SDNS/FRNS. (**A**) Expression level of HLA-DQA1 detected using flow cytometry (left). Percentages of cells in the relapse and remission stages (right). (**B**) Expression level of HLA-DQB1 detected using flow cytometry (left). Percentages of cells in the relapse and remission stages (right). (**C**) mRNA levels of HLA-DQA1. (**D**) mRNA levels of HLA-DQB1 (*p < 0.05, **p < 0.01)

To detect HLA-DQA1 and HLA-DQB1 in the disease group (relapse and remission stages) and healthy controls, we performed multiparametric flow cytometry on PBMCs. Among the groups, the lowest proportion of cells expressing HLA-DQA1^+^/HLA-DQB1^+^ occurred in the relapse stage (HLA-DQA1^+^, *P* = 0.0046; HLA-DQB1^+^, *P* = 0.0045) (Fig. 2A). After treatment, the proportion of cells expressing HLA-DQA1^+^/HLA-DQB1^+^ increased in the remission stage. We also observed no difference in the expression of HLA-DQA1 or HLA-DQB1 between patients in the remission stage and healthy controls (Fig. 2B). Analysis of the qRT‒PCR results revealed that the mRNA levels of *HLA-DQA1* and *HLA-DQB1* were significantly greater in the relapse group than in the remission group (*HLA-DQA1*, *P* = 0.03; *HLA-DQB1*, *P* = 0.002), suggesting that HLA-DQA1 and HLA-DQB1 affect disease relapse in SDNS/FRNS patients (Fig. 2C‒D).

### Association between the genotype at rs1047989 and the expression of HLA-DQA1 in SDNS/FRNS patients

**Fig. 3:**
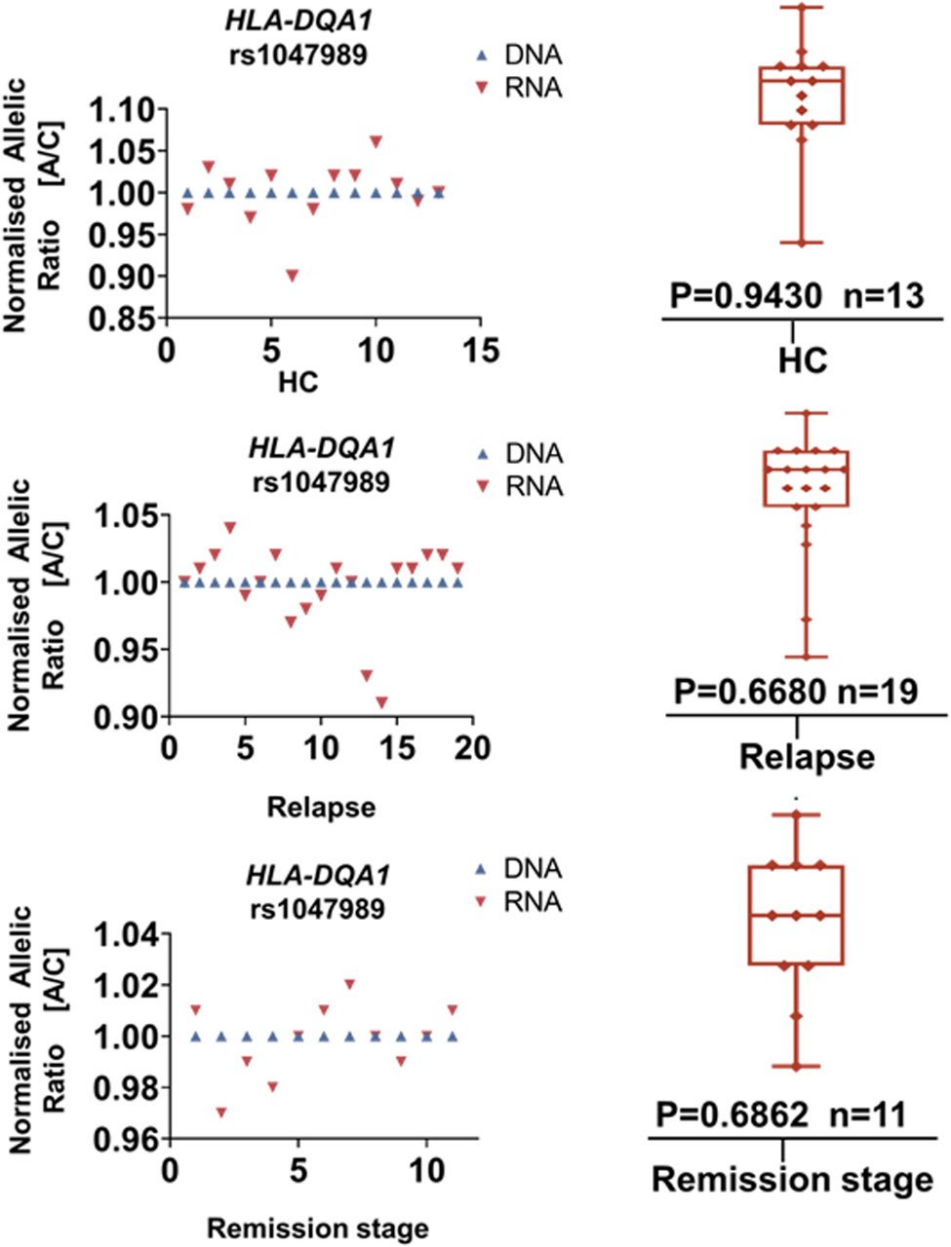
AEI analysis of *HLA-DQA1* in different stages of SDNS/FRNS. (**A**) Allelic ratios for the *HLA-DQA1* transcript SNP rs1047989 (A/C; A = SDNS/FRNS risk allele) in healthy controls. (**B**) Allelic ratios for the HLA-DQA1 transcript SNP rs1047989 (A/C; A = SDNS/FRNS risk allele) in the relapse stage of SDNS/FRNS. (**C**) Allelic ratios for the HLA-DQA1 transcript SNP rs1047989 (A/C; A = SDNS/FRNS risk allele) in the remission stage of SDNS/FRNS. *P* values were calculated using Wilcoxon’s matched pairs signed rank test

rs1047989 resides within the open reading frame region of *HLA-DQA1*, which encodes the HLA-DQA1 complex that is closely involved in the activation of professional cells. In this study, we discovered that the risk allele of rs1047989 is associated with a greater number of infections at relapse in SDNS/FRNS patients. Therefore, we undertook an AEI analysis in peripheral blood samples from SDNS/FRNS patients to assess whether the rs1047989 genotype was associated with the expression of HLA-DQA1 in these patients. None of the groups demonstrated AEI (Fig. 3), although in SDNS/FRNS patients, the risk allele A of the *HLA-DQA1* transcript SNP rs1047989 showing an average 0.999-fold increase in healthy controls (*P* = 0.94), an average 0.997-fold increase in the relapse group (*P* = 0.67), and an average 0.998-fold increase in the remission group (*P* = 0.68). Since the risk variants of HLA-DQB1 are located in the intron region, AEI analysis was not performed.

### Nonsynonymous variants at rs1047989 were predicted to significantly affect the HLA-DQA1 protein

**Fig. 4:**
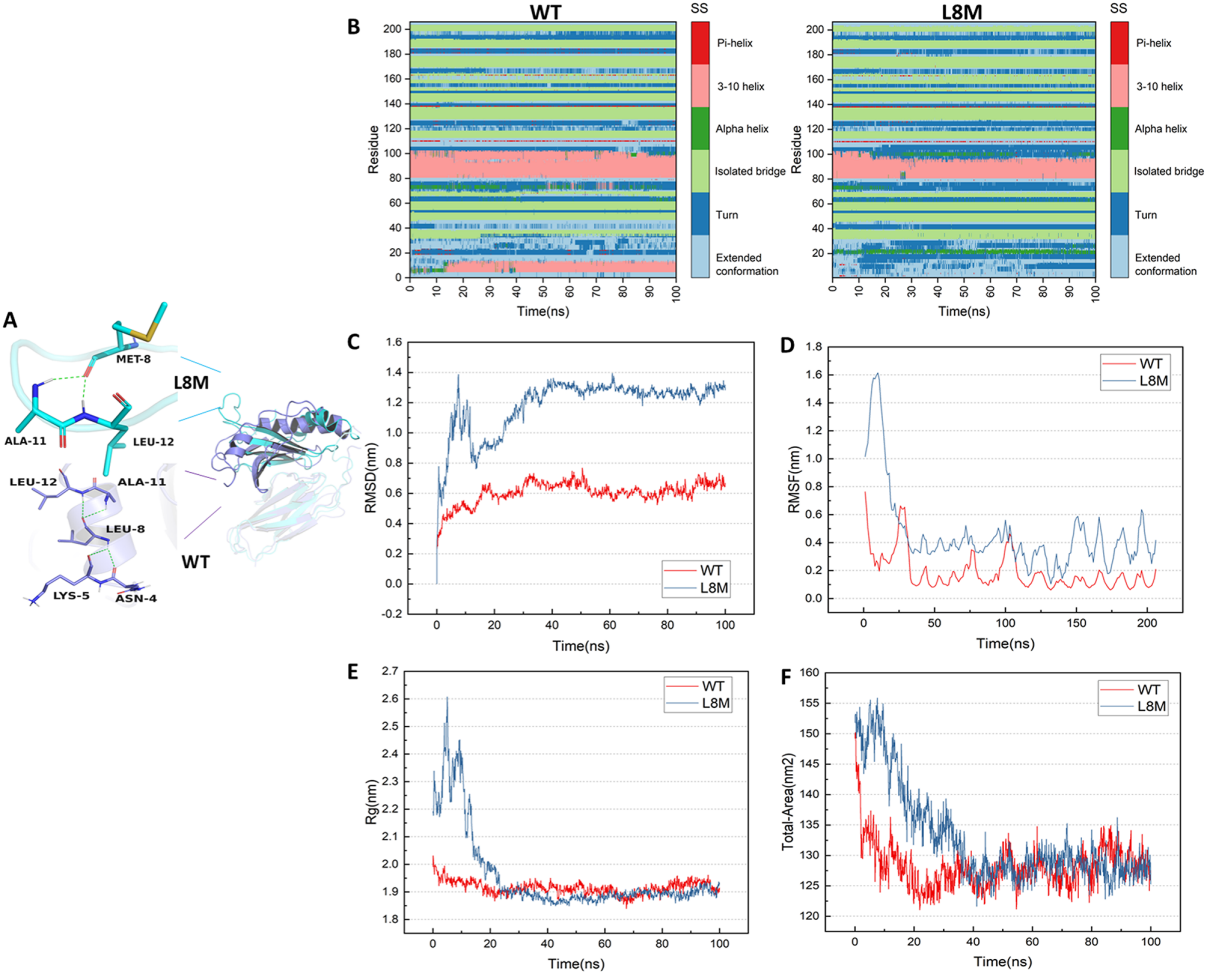
Protein structure modeling and molecular dynamic measurement of rs1047989 in *HLA-DQA1*. (**A**) Structures of the L8M and WT proteins. (**B**) Secondary structures of the L8M and WT proteins. (**C**) RMSD used to measure the ligand stability of the L8M and WT proteins. (**D**) Structural fluctuations of local amino acids. (**E**) Evaluation of the tightness of the architecture. (**F**) Measurement of the surface area of HLA-DQA1. L8M, mutant amino acid change in HLA-DQA1; WT, wild-type HLA-DQA1; RMSD, root-mean-square deviation; RMSF, root-mean-square fluctuation; Rg, radius of gyration; SASA, solvent accessible surface area

The genetic and clinical significance of rs1047989 has been confirmed, and we conducted structural modeling and molecular dynamic measurements. The rs1047989 locus is a transcript SNP that introduces amino acid (nonsynonymous) substitutions into HLA-DQA1 (L8M). The structures of the L8M and WT proteins at 100 ns showed significant changes in the N-terminal domain, whereas the C-terminal domain showed slightly smaller changes (Fig. 4A). This finding indicates that the variant is likely to affect the structure or stability of HLA-DQA1. The secondary structure of residues within 100 ns was further analyzed. The L8M protein has a greater probability of extended conformation and turn structures. The probability of an α2-helix with a residue number of approximately 100 in the L8M protein was greater than that in the WT protein (Fig. 4B). The RMSD was used to measure ligand stability during simulation, and the overall structural changes in the L8M protein were greater than those in the WT protein (Fig. 4C). The RMSF can be used to detect the structural fluctuations of local amino acid residue sites in the system during the simulation process. Our study revealed that, for the L8M protein, the overall residue changes were significant, especially between residues 0 and 50 (Fig. 4D). Rg is used to evaluate the tightness of the architecture. Our study confirmed that the compactness of the L8M protein system significantly decreased between 0 and 40 ns (Fig. 4E). The SASA is a parameter used to measure the surface area of proteins in contact with solvents. In our study, the overall hydrophilic surface area of the WT protein was greater than that of the L8M protein, and the overall hydrophobic surface area of the L8M protein was greater than that of the WT protein (Fig. 4F). In addition, we analyzed the hydrogen bonds formed in the protein‒ligand systems, and both systems remained stable between 120 and 140 ns.

## Discussion

Most children with SSNS respond to a standard course of oral corticosteroids. However, most patients tend to have experience disease relapse throughout childhood. The disease course varies, with 35–40% of patients having a single episode or one to two relapses and 55–60% having more frequent relapses [15]. We defined patients with a single relapse episode as having SSNSWR and patients with SDNS/FRNS in this study. To date, the pathophysiology of SSNS in childhood is not clearly understood. However, it is considered a result of the interaction between genetic risk and environmental factors [16–19]. Several previous studies have reported strong associations between genetic loci in the HLA regions and SSNS in different populations [20–21]. We confirmed previous findings and described several high-risk variants and genes associated with the two main subphenotypes of SSNS [9]. In this study, we further analyzed the clinical significance of these high-risk variants and the molecular roles of HLA-DQA1 and HLA-DQB1 during the disease period. Considering that non-invasive methods such as genetic analysis and molecular diagnostic techniques are gradually being used as auxiliary diagnostic tools, our study can provide more precise information enabling the individualized treatment of SSNS patients.

The replication study included an independent population comprising 206 Chinese participants from the Children’s Hospital of Chongqing Medical University. Significant differences in the frequency of allele A at rs1464545187 in *ANKRD36* between SSNSWR patients and healthy controls were detected. *ANKRD36* is located on chromosome 2, has 36 exons and contains 6 repeat units. There are few studies on *ANKRD36*, and most of them focused on GWASs. ANKRD36 mutants and high ANKRD36 expression were also found to be associated with tumors [22–24]. In the field of nephrology, ANKRD36 was found to be involved in blood pressure regulation by interacting with transcriptional repressor proteins and subsequently altering the expression of epithelial sodium channel genes [25]. Pan et al. [26] reported that *ANKRD36* may be involved in the progression of diabetic kidney disease through lipid metabolism and inflammation. However, the underlying mechanism of *ANKRD36* in renal damage is still unclear. In patients with pneumonia and myocarditis [27–28], ANKRD36 plays a proinflammatory role by regulating intracellular NF-κB inflammation-related pathways. We hypothesize that the ANKRD36 protein regulates lipid metabolism through protein interactions in SSNS patients. Considering that ANKRD36 is a new inflammatory marker for SSNS, future studies should explore whether *ANKRD36* regulates immune response status.

In recent years, genetic studies have provided evidence that human HLA histocompatibility plays a causal role in the disease course of SSNS. HLA histocompatibility is a crucial component of the human immune system and is encoded by a complex of genes located on the short arm of chromosome 6. These antigens are expressed by the major histocompatibility complex (MHC), which plays a pivotal role in determining tissue compatibility and rejection reactions during transplantation. HLAs are categorized into three classes according to their distribution and function: Class I, Class II, and Class III. The classical class I antigens include HLA-A, HLA-B, and HLA-C, whereas class II antigens include HLA-DP, HLA-DQ, and HLA-DR. Among the classical HLA variants, our study revealed that 3 variants (rs1047989, rs9273471, and HLA-DQB1*06:02) from the HLA-DQA/DQB region are significant and are major determinants of childhood SDNS/FRNS. In addition, we confirmed that these HLA variants have several important biological functions and can affect disease progression. For example, rs1047989 in *HLA-DQA1* was significantly associated with a greater number of infections at relapse in SDNS/FRNS patients and with the differential expression of HLA-DQA1 and HLA-DQB1 in the relapse and remission stages of SSNS. These findings provide new insights into our understanding of the gene‒environment interaction in childhood SSNS and further support an infective and immunologic basis for its pathogenesis. Consequently, our principal findings are that the HLA-DQA1 or HLA-DQB1 complex is a critical entity that underlies the relationship between immunological reactions and the risk of developing SDNS/FRNS, which has a strong likelihood of being stimulated by autoimmunity or infection.

In our study, in addition to examining the expression of *HLA-DQA1* and *HLA-DQB1* in the relapse and remission stages of SSNS patients, we revealed that rs1047989, a susceptibility functional variant of *HLA-DQA1*, had a strong effect on overall HLA-DQA1 expression via cis-expression quantitative trait loci (eQTLs). For patients with SDNS/FRNS in the relapse or remission stage, AEI was not detected, suggesting that the risk allele A of rs1047989 was not associated with decreased HLA-DQA1 expression. Compared with the wild-type allele, the risk allele A of rs1047989 does not have preferential expression advantages during the transcription process. Unlike other non-coding SNPs, the rs1047989 locus encompasses a transcript SNP that introduces amino acid (nonsynonymous) substitutions. To determine the reasons for the decreased expression of HLA-DQA1, we performed protein structure modeling and molecular dynamics for rs1047989. Interestingly, this variant is highly likely to affect the structure or stability of HLA-DQA1. These alterations in structure or stability may contribute to the reduction in HLA-DQA1 in the relapse stage in patients with SDNS/FRNS. A published study suggested that single amino acid changes in one HLA protein may play a key role in HLA function by altering the local 3-dimensional conformation of certain HLA molecules [29]. These results can be explained by two mechanisms. One possible mechanism is that genes of the HLA class II protein complex and HLA class II receptor activity are strongly associated with the innate immune system, including the activation of professional cells (antigen-presenting cells and B cells), as well as the adaptive immune system, in which HLA molecules play a crucial role in the disease process [30–31]. Another potential mechanism is that polymorphisms of HLA-DQA1 amino acids may affect T helper cell regulation and amplify the immune response downstream by changing the binding affinity with antigen peptides.

## Conclusion

In conclusion, in the present study, we identified, for the first time, the genetic associations of HLA-DQA1 and HLA-DQB1 with SSNS at the transcription and translation levels; in addition, we found that the specific amino acid polymorphisms in these molecules are major determinants that can be used to explain the relapse status of patients with childhood SDNS/FRNS. These results suggest that HLA-DQA1 and HLA-DQB1 play pivotal roles in SSNS pathophysiology. Our study could help prioritize the epidemiology and prevention of SDNS/FRNS and motivate the development of future studies to provide further insight into the mechanisms underlying childhood SSNS.

## Electronic supplementary material

Below is the link to the electronic supplementary material.

**Supplementary Material 1**: **Supplementary Table 1**. Process of genomic DNA extraction. **Supplementary Table 2**. Primers for polymorphic loci. **Supplementary Table 3**. Primer sequences used for real-time PCR.

## Data Availability

All data generated or analysed during this study are included in this published article and its supplementary information files.

## Abbreviations

INS: Idiopathic nephrotic syndrome
SSNS: Steroid-sensitive nephrotic syndrome
SDNS/FRNS: Steroid-dependent and (or) frequent relapse nephrotic syndrome
SSNSWR: Steroid-sensitive nephrotic syndrome without relapse
AEI: Allele expression imbalance
CI: Confidence interval
OR: Odds ratio
GWAS: Genome wide association studies
PBMCs: Peripheral blood mononuclear cells
qRT-PCR: Quantitative reverse-transcription polymerase chain reaction
MD: Molecular dynamic
RMSD: Simulation results such as root-mean-square deviation
RMSF: Root-mean-square fluctuation)
Rg: Radius of gyration
SASA: Solvent accessible surface area
DSSP: Dictionary of the secondary structure of protein
GAD1: Glutamic acid decarboxylase 1
ANKRD36: Ankyrin Repeat Domain-Containing Protein 36
