# Supplementary material for: Amino acid variants in the HLA-DQA1 and HLA-DQB1 molecules explain the major association of variants with relapse status in pediatric patients with steroid-sensitive nephrotic syndrome
Source: Ital J Pediatr. 2025 Mar 14;51:79. doi: 10.1186/s13052-025-01913-z (PMC11909919; doi:10.1186/s13052-025-01913-z)
Supplement: Supplementary file 1 — Supplementary Material 1: Supplementary Table 1. Process of genomic DNA extraction. Supplementary Table 2. Primers for polymorphic loci. Supplementary Table 3. Primer sequences used for real-time PCR. [file 13052_2025_1913_MOESM1_ESM.docx]

Supplementary Information

**Amino acid variants in the HLA-DQA1 and HLA-DQB1 molecules explain the major association of variants with relapse status in pediatric patients with steroid-sensitive nephrotic syndrome**

Supplementary Table 1. Process of genomic DNA extraction.

Supplementary Table 2. Primers for polymorphic loci.

Supplementary Table 3. Primer sequences used for real-time PCR.

Supplementary Table 1. Process of genomic DNA extraction.

| Step | Experimental operation |
| --- | --- |
| 1 | Pipet 20µl QIAGEN Protease stock solution into the bottom of the collection microtubes. |
| 2 | Add samples to the collection microtubes by touching the insides of the tubes without wetting the rims. Use either 200µl whole blood, plasma, serum, or body fluids per tube, or up to 5x106 lymphocytes or cultured cells in 200µl PBS per tube. Use the Plate Register provided to record the locations of the samples. |
| 3 | Add 200µl Buffer AL to each sample, taking care not to wet the rims of the collection microtubes. Seal the tubes using the caps for collection microtubes provided. |
| 4 | Cover the rack with the plastic cover supplied, and mix thoroughly by shaking vigorously for 15s. |
| 5 | Centrifuge briefly at 3000 rpm to collect any solution from the caps. |
| 6 | Incubate at 70°C for at least 10 min in an incubator or oven. |
| 7 | Centrifuge briefly at 3000 rpm to collect any lysate from the caps. |
| 8 | Remove the caps and add 200µl ethanol (96-100%) to each tube. |
| 9 | Seal the tubes using new caps for collection microtubes. Shake vigorously for 15s. |
| 10 | Centrifuge briefly at 3000 rpm to collect any solution from the caps. |
| 11 | Carefully apply the mixture from step 8 (620µl per collection microtube) to the QIAamp 96 plate. |
| 12 | Seal the QIAamp 96 plate with an AirPore Tape sheet. Load the S-Block and QIAamp 96 plate onto the carrier, then place it in the rotor bucket. Centrifuge at 6000 rpm for 4 min. |
| 13 | Remove the tape. Carefully add 500µl Buffer AW1 to each well. |
| 14 | Seal the QIAamp 96 plate with a new AirPore Tape sheet. Centrifuge at 6000 rpm for 2 min. |
| 15 | Remove the tape. Carefully add 500µl Buffer AW2 to each well. |
| 16 | Centrifuge at 6000 rpm for 15 min. |
| 17 | Place the QIAamp 96 plate on top of a rack of elution microtubes (provided). |
| 18 | To elute DNA, add 200µl Buffer AE or distilled water, equilibrated to room temperature, to each well using a multichannel pipet. Seal the QIAamp 96 plate with a new AirPore tape sheet and incubate for 1 min at room temperature. Centrifuge at 6000 rpm for 4 min. Seal the wells of the microtubes for storage using the caps for elution microtubes provided. |

Supplementary Table 2. Primers for polymorphic loci.

| Target gene | Primer sequence |
| --- | --- |
| HLA-DQB1*06:02 | forward 5′‐CCCAGGAAATGCTTCTCCACAG‐3′ |
|  | reverse 5′‐GGATTGGATGGTCCCTCGGAA‐3′ |
| chr2:171713702 | forward 5′‐GACTGGAGTGCCTTTGAAAGAGG‐3′ |
|  | reverse 5′‐CATGCCCAGCCTGAAAAACAC‐3′ |
| rs1049123 | forward 5′- TTCACCTTTGCACAGATCTTGGGG-3′ |
|  | reverse 5′- TACACAGCACTCACCAAACCAGAA-3′ |
| rs9273471 | forward 5′- CTTGTGAAATGTGGAGCCAGAACC -3′ |
|  | reverse 5′- AAGAATAGAAACAGAAACCCCTTGGG -3′ |
| rs1464545187 | forward 5′- TTTCCCACAAGAGACTTTGCAGGG -3′ |
|  | reverse 5′- AAATTTGGGGATTGTTCAGGCCT -3′ |
| rs1047989 | forward 5′- ACTCTGAGTAGAGGCTGCATCAC-3′ |
|  | reverse 5′- TTGAGGCAATCATGATGGAACGAC -3′ |
| rs117962550 | forward 5′- AGGATGCACACTGCACTATACCC -3′ |
|  | reverse 5′- GTCCATGGCCAGGAAGGTCT -3′ |

Supplementary Table 3. Primer sequences used for real-time PCR.

| Target mRNA | Primer Sequence |
| --- | --- |
| HLA-DQA1 | forward 5′‐GCTGTGGCAAAACACAACTTGAACA‐3′ |
|  | reverse 5′‐CTGTGACTGACTGCCCATTGCT‐3′ |
| HLA-DQB1 | forward 5′‐ATGTCTTGGAAGAAGGCTTTGCG‐3′ |
|  | reverse, 5′‐TCCGTCCCGTTGGTGAAGTAGC‐3′ |
| GAPDH | forward 5′‐GCACCGTCAAGGCTGAGAAC‐3′ |
|  | reverse, 5′‐ATGGTGGTGAAGACGCCAGT‐3′ |
